# Chemical-Sensory Traits of Fresh Cheese Made by Enzymatic Coagulation of Donkey Milk

**DOI:** 10.3390/foods9010016

**Published:** 2019-12-23

**Authors:** Michele Faccia, Giuseppe Gambacorta, Giovanni Martemucci, Graziana Difonzo, Angela Gabriella D’Alessandro

**Affiliations:** 1 Department of Soil, Plant and Food Sciences, University of Bari, Via Amendola 165/A, 70126 Bari, Italy; michele.faccia@uniba.it (M.F.); graziana.difonzo@uniba.it (G.D.); 2 Department of Agro-Environmental and Territorial Sciences, University of Bari, Via Amendola 165/A, 70126 Bari, Italy; giovanni.martemucci@uniba.it (G.M.); angelagabriella.dalessandro@uniba.it (A.G.D.)

**Keywords:** cheesemaking, donkey milk, fatty acids, sensory analysis, VOC

## Abstract

Making cheese from donkey milk is considered unfeasible, due to difficulties in coagulation and curd forming. Two recent studies have reported the protocols for making fresh cheese by using camel chymosin or calf rennet, but the chemical and sensory characteristics of the products were not thoroughly investigated. The present paper aims to give a further contribution to the field, by investigating cheesemaking with microbial rennet and evaluating the chemical composition, total fatty acid, volatile organic compounds (VOCs) and sensory profile of the resultant product. Six trials were undertaken at laboratory scale on donkey milk from a Martina Franca ass, by applying the technological scheme as reported for calf rennet, with some modifications. Bulk cow milk was used as a control. Donkey milk coagulated rapidly, but the curd remained soft, and was only suitable for making fresh cheese; differently, cow milk coagulated almost instantaneously under these strong technological conditions, giving rise to a semi-hard curd in very short time. The moisture level of donkey cheese was almost the same as reported in the literature, whereas the yield was higher, probably due to the high protein content of the milk used. The total fatty acid composition of cheese presented some differences with respect to milk, mostly consisting in a higher presence of saturated compounds. A connection with a better retention of the large sized fat globules into the curd was hypothesised and discussed. The VOC analyses, performed by solid-phase micro extraction gas chromatography-mass spectrometry, allowed the identification of 11 compounds in milk and 18 in cheese. The sensory characteristics of donkey cheese were strongly different with respect to the control, and revealed unique and pleasant flavours.

## 1. Introduction

The use of equid milk for human nutrition and wellness has an ancient history. The use of mare milk for producing koumiss, a lactic–alcoholic beverage, belongs to the food tradition of several ethnic groups from Central Asia [1]. Ancient Romans and Greeks were already aware of the cosmetic and therapeutic properties of donkey milk, as evidenced by the legendary milk bath of Cleopatra and Poppea, and Hippocrates’ indications as a remedy for several diseases [2,3]. After a long and slow decline, donkey rearing is now undergoing a revival, due to an interest in innovative and nutraceutical foods. Donkey milk is not in competition with cow’s milk, and represents an added value functional food with high market potential. Besides, European policy encourages the diversification of agricultural production and the valorisation of local genetic resources. Donkeys are able to use marginal areas unsuitable for specialised dairy cows, and they are better adapted to warm climates. 

For these reasons, rearing donkeys for milk is gaining increasing attention in the Mediterranean area, in particular Italy [4], where milk is sold as a raw product directly at farms. Other forms of marketing at niche level also exist, such as selling online or through pharmacies as pasteurised or, rarely, as UHT-treated or freeze-dried product [5]. The interest of the food researchers towards donkey milk is also increasing; several protocols for manufacturing innovative fermented milks and functional beverages at laboratory level are already available [6,7,8]. Unfortunately, studies on cheesemaking are very rare, since rennet coagulation is commonly considered as unfeasible. Apart from economic considerations connected to the high cost and low availability of the raw matter, the main technical obstacles seem to be the low casein concentration (0.64 to 1.03 g/100 g milk) and different micelle structure with respect to ruminants [9]. Recent studies have reported that donkey casein micelles are much larger than bovine micelles (298.5 ± 18.9 nm at pH 6.8 versus 186.5 ± 1.2 nm), have a lower absolute zeta potential (−15.4  ±  0.5  mV), poor colloidal stability and different abundance of the casein fractions [10,11]. This latter aspect has long been debated, until the work of Chianese et al. [12] definitively clarified the matter using electrophoresis and immunostaining of the protein bands with polyclonal antibodies against the four ‘classic’ caseins. The authors ascertained the presence of κ-casein, and demonstrated that β-casein was the most abundant fraction, followed by αs1 and αs2.

Despite the difficulty in milk coagulation, some successful efforts in making fresh donkey cheese have been recently reported. In particular, Iannella [13] and Faccia et al. [14] developed two different cheesemaking protocols, based on the use of camel chymosin or calf rennet, respectively. Even though the results obtained were encouraging, the two studies had some conflicting points, and the products were only poorly investigated. The aim of the present study was to provide a more in-depth insight on the chemical and sensory traits of a cheese made from donkey milk by using microbial rennet.

## 2. Materials and Methods

### 2.1. Milk and Cheesemaking Trials

**Figure 1:**
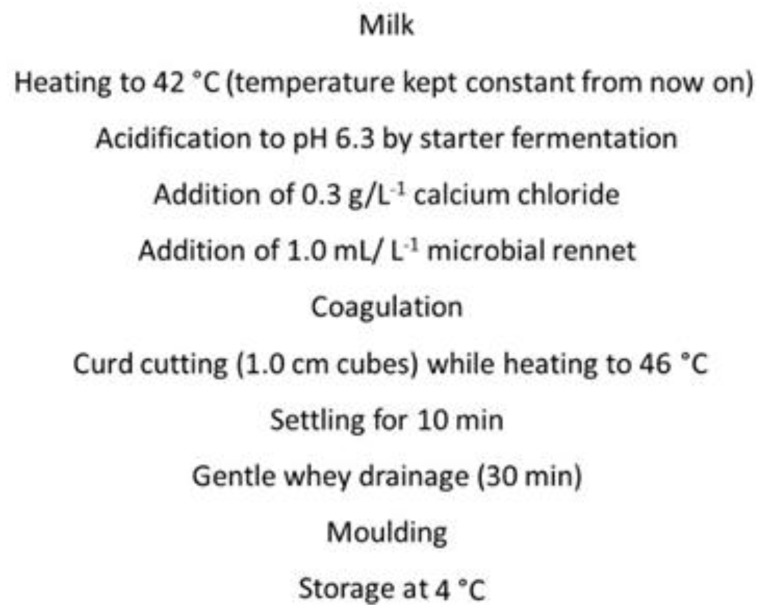
Technological scheme used for donkey milk cheesemaking.

Milk was derived from 10 donkeys (Martina Franca ass) at the mid-period of lactation routinely milked by a mechanical milker at a farm located in the Apulia region (Southern Italy). The gross composition (fat, protein and lactose) was assessed by Infrared Spectroscopy by MilkoScan™ FT1 (FOSS, Hillerød, Denmark). Cheesemaking with donkey milk, using bulk cow milk as the control, was carried out at laboratory level (six replicates) by following the protocol reported by Faccia et al. [14] with some modifications (Figure 1). The experimentation was performed in small pots using 5 L milk, which was heated and maintained at the desired temperatures in a thermostatic water bath. The starter used was *Streptococcus termophilus* (Choozit Star, Danisco/Dupont, Cernusco sul Naviglio, Italy), whereas microbial rennet was Fromase from *Rhizomucor miehei* (normal strength, DSM, Delft, The Netherlands). The curd cutting time was established by using a vibro-viscometer (SV-10; A & D Company, Limited, Tokyo, Japan) applied to a 40 mL aliquot acidified and renneted milk sample, taken from the pot and kept at 42 °C. Cutting started when the viscosity value did not change within 3 min; the control milk coagulum was also cut at the same time. After moulding, samples of whey were taken for determination of the fat content (Gerber method) and of the total fatty acids profile. The cheeses obtained, weighing about 300 g, were kept at 25 °C for 2 h, and then were dry salted and stored overnight at 4 °C. The following morning (about 24 h after production), they were weighted for yield calculation and subjected to chemical and sensory analyses.

### 2.2. Chemical and Sensory Analyses

The moisture content in cheese was assessed by the method IDF 4:1986, fat by the Soxhlet method, total protein by the Kjeldhal method, ash by muffle furnace incineration at 530 °C, and pH by the HI99165 pH Meter equipped with penetration electrode (Hanna Instruments, Woonsocket, RI, USA). Total fatty acids (TFA) analyses were performed on milk, whey and cheese by gas chromatography (GC), as reported by Faccia et al. [15], with some modifications. Briefly, the fatty acids methyl esters were obtained by transesterification of about 25 mg lipid sample obtained by Soxhlet extraction (for cheese) and centrifugation at 2.360 RCF at 4 °C (for milk and whey), previously dissolved in 2 mL of petroleum ether. Samples were injected into a Fisons model MFC 800 gas chromatograph (Fisons, Milan, Italy) equipped with a 60 m × 0.32 mm i.d. and 0.5 μm film thickness fused-silica capillary column (Stabilwax, Restek, Bellefonte, PA, USA). The operating conditions were: (a) oven temperature 70 °C for 5 min, then to 220 °C at 1 °C/min, and held at 220 °C for 30 min; (b) carrier gas helium 20 cm/s at 170 °C; (c) injector at 250 °C, 1 μL; split 40:1; (d) flame-ionisation detector, 250 °C. Volatile compounds were extracted by the HS-SPME technique using a DVB-CAR-PDMS fiber (Supelco, Bellefonte, Pa., USA), as reported by Gambacorta et al. [16], with some modifications. The sample (1.0 mL milk or 1 g cheese), 0.2 g sodium chloride and 10 L of 3-pentanone (internal standard, 81.9 ng/L in water) were placed in 20 mL screw-cap vial, tightly capped with a PTFE-silicon septum, and conditioned for 10 min at 40 °C. A TriPlus RSH™ Autosampler (Thermo Fisher Scientific, Rodano, Italy) was used to optimise and standardise the entire extraction procedure. The fiber then was introduced into the headspace of the vial for 40 min at 40 °C. Then, desorption of volatiles from fiber took place in a splitless mode for 3 min at 220 °C. The separation of volatile compounds was performed by a Trace 1300 gas chromatograph (Thermo Fisher Scientific) equipped with a VF-WAXms capillary column (Agilent, Santa Clara, CA, USA), 60 m length × 0.25 mm I.D. × 0.25 μm film, and coupled with an ISQ single quadrupole mass spectrometer (Thermo Fisher Scientific). The chromatographic conditions were: oven, 45 °C (5 min) to 210 °C at 4 °C/min, held for 3 min; detector, source temperature 250 °C; transfer line temperature 250 °C; carrier gas, helium at constant flow of 0.4 mL/min. The impact energy was 70 eV. Data were acquired using the full-scan mode in the range of 35 to 150 m/z at an acquisition rate of 7.2 Hz. Volatile compounds were tentatively identified by comparing the experimental spectra with those reported in the National Institute of Standards and Technology (NIST) Library, and with those obtained by the available pure standard compounds. Semi-quantitation was carried out by considering the relative areas of the compounds related to that of the internal standard, and assuming arbitrarily that their response factors were the same. 

Acquisition and processing of peaks was carried out using Xcalibur v 4.1 software (Thermo Fisher Scientific). All analyses were done in triplicate, and means and standard deviations were calculated. Two different electrophoresis (EF) techniques were used for investigating the protein fraction in milk, whey and cheese. A sample of isoelectric whey, used as a standard, was also prepared. The milk and cheese samples were analysed by urea polyacrylamide gel electrophoresis (PAGE) as indicated by Andrews [17], whereas sodium dodecyl sulphate (SDS) PAGE as reported by Harper et al. [18] was applied to the whey samples. Identification of the protein bands was done by comparison with the data reported by Egito et al. [19,20] and Chianese et al. [12], and for SDS PAGE, the molecular weight was also taken into consideration.

For sensory analysis, the Flavour Profile Method (FPM) was applied, since the product was very innovative, and derived from an “uncommon” raw matter. In fact, due to lack of familiarity, during the training sessions the assessors familiarised with a sample of raw donkey milk before approaching the cheese. Even though FPM can suffer of possible influences among assessors during the final discussion, the analysis is easier to perform than the other descriptive methods [21,22]. It requires a restricted group of trained panellists (at least four) and the generation of a group consensus profile. A trained panel composed of five members (two males and three females aged 28–56 years with three years’ experience in cheese analysis) coordinated by a panel leader, performed the evaluation. The identification and selection of descriptors was carried out according to the ISO 11035 protocol [23]. The panel had two open sessions for approaching the new product, during which the cheese was tested by smelling, touching and tasting. A first list of descriptive terms was developed, and then the list was reduced according to the value of frequency of citations × the perceived intensity [24]. For quantitation, a series of reference products were chosen, on which the panel performed three training sessions. Finally, two sessions (in duplicate) were used for developing the qualiquantitative profile by adopting a nonstructured scale. By collegial discussion, a final graph was developed in which the attributes appeared in temporal order (from left to right, from the first to the last perceived, respectively) and their intensity was expressed by a vector of different length.

## 3. Results and Discussion

**Figure 2:**
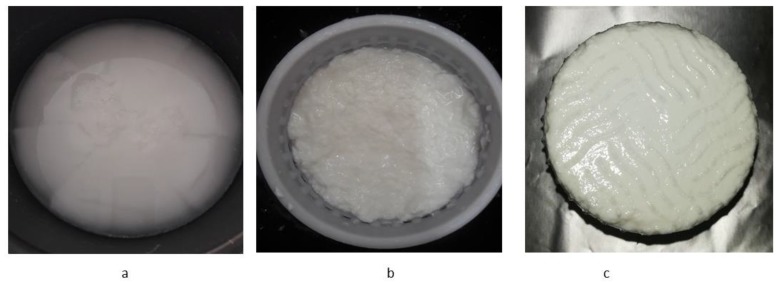
Donkey cheese manufacturing: curd at the cutting time (**a**); moulded curd (**b**); cheese after 24 h (**c**).

The adopted protocol allowed rapid milk coagulation, since the time from rennet addition to curd cutting was 9.15 ± 3.10 min. However, as already reported for calf rennet [14], the coagulum was very soft, and gentle processing was needed to obtain a curd with sufficient firmness, suitable to be moulded (Figure 2). The process consisted of cutting the curd by knife, heating to 46 °C and keeping constant the temperature while progressively removing the whey. On the average, this phase required more than 30 min, and the total processing time (until moulding) was 42.10 min. The cheese texture remained soft and slightly compacted after 24 h. Differently (and as expected), when applied to cow milk, the same technological conditions caused immediate coagulation, very firm curd and intense syneresis, with a total processing time of about 13 min. The results of the present experimentation, together with those previously reported for the use of calf rennet [14], suggest that any type of rennet allows curd formation, if used under suitable conditions. These findings refute the hypothesis of Iannella [13], according to which only camel chymosin can be used for making donkey cheese. Differently, it was evident that the most relevant difficulty does not lie in the primary phase of coagulation, but in the secondary one, which is mainly driven by ionic calcium, heat and casein concentration. In this study, fortification with ionic calcium (CaCl_2_) and heating were applied at the same level, as in the previous study on calf rennet (0.3 g/L^−1^ and to 46 °C, respectively; [14], since increasing them to 0.4 g/L^−1^ and 50 °C had no effect.

Table 1 shows the characteristics of the donkey cheese in comparison with the control one manufactured from cow milk. As expected, the processing time, yield and chemical composition were very different. For donkey, the processing time was more than three times longer and yield was more than three times lower. As to the chemical composition, it was very close to be a fat-free product, and the moisture content was typical of fresh, soft cheeses; the control was semi-hard.

The total solids contentd of donkey cheese was close to those reported in the literature [13,14], whereas the yield was much higher. This latter result was probably connected to the high protein content of the milk, but the impact of microbial rennet should be investigated further. In fact, since all caseins contribute to the colloidal stability of the micelle (κ-fraction is poorly present in donkey milk), the typical, nonspecific, proteolytic activity of microbial coagulant could have some relevance [25,26]. As regards the total processing time, it was not very different from that reported when using calf rennet, but much shorter with respect to that when camel chymosin was applied. Such great difference could indicate that this latter product was not obtained by ‘pure’ enzymic coagulation, but by ‘combined enzymic-acid’ technology. In fact, the total processing time reported for camel chymosin (more than 5 h) very likely allowed acidification by the starter microflora added, which contributed to coagulation and curd formation.

TFA analyses of milk, whey and cheese samples were carried out in order to evaluate if the fatty acid profiles remained unchanged after cheesemaking. Table 2 shows the results obtained. As to donkey milk, the most abundant compound was oleic acid, followed by palmitic, linoleic, capric and lauric. 

The results agree with those obtained by Martemucci and D’Alessandro [27] in samples taken at 90 days of lactation. Some differences were observed with the data of Gastaldi et al. [28], who reported a different order of abundance. This discrepancy could be connected to a different lactation stage; however, the authors did not indicate the period in which they took the samples. The total amount of saturated fatty acids was very close to that found by Martini et al. [29] in milk from the Amiata donkey, whereas the concentration of polyunsaturated was much higher. The average TFA profile of the bovine milk used as control (bulk milk samples taken from an industrial dairy) was within the typical range of this type of milk, and the high saturated/polyunsaturated ratio suggests possible derivation from intensive farms. The TFA profile of the cheese showed some changes with respect to milk, due to an increase of some saturated compounds (in particular stearic acid) and decrease of the polyunsaturated ones (mainly linoleic and linolenic acids). This result was surprising, and was only partially reflected by the control, for which the differences were less pronounced and the significance was only found for stearic acid. At our knowledge, this argument has been poorly investigated, and more analyses are needed to confirm the result. A possible cause could lie in a different retention of the fat globules into the cheese, depending on their size. According to the literature, small sized and large sized globules of cow milk present some differences in the fatty acid composition of glycerides: the small globules contain less short chain fatty acids, less stearic and more oleic acid [30,31] than larger ones. For donkey milk, no information is available on TFA composition of fat globules glycerides, but Martini et al. [29] reported that globules have different size, ranging from less than 2 to more than 5 µm, and that the small ones are much more abundant (about 70% of total). If, as theoretically expected, the small globules are less retained into the curd, some differences in the TFA profile of milk, cheese and whey should arise. Unfortunately, the TFA analyses of the whey samples did not give further elements for supporting this hypothesis, since the standard deviation was too high. A specific study is required to in order to clarify the matter.

The volatile organic compounds (VOCs) analyses gave the results shown in Table 3, in which only the compounds detected in at least four trials (out of six) are shown. Information on VOCs in donkey milk is rare: Conte et al. [32], Tidona et al. [33] and Vincenzetti et al. [34] identified 7, 6 and 19 compounds, respectively. In our study, 11 individual volatile compounds were identified (versus 21 in the control), and the total peak area was much lower than in cow milk. The different fat content in the two types of milk should play a role in the formation and release of VOCs. In this regard, it must be underlined that the results on the donkey samples (both milk and cheese) had low standard deviations, differently from those obtained in the cow cheese. It could be connected to the different concentration at which VOCs are present in the two types of samples, or to the variability of the fat content (3.62 ± 0.39) in the cow milk samples used. The most abundant volatile compounds in donkey milk were acetic acid and acetone: they can both derive from animal metabolism and microbial activity. These two compounds were also detected in cheese, but at a lower level, probably because they were lost into the whey during cheesemaking. Among the other compounds, 6-methyl-5-hepten-2-one was the third in order of abundance: it is commonly called sulcatone, and presents an apple, bitter and citrus flavour. It is a powerful attractant for mosquitos, and is emitted by a variety of plants and animals, including humans, horses, cows and sheep. Sulcatone is also present in the faeces of mares in estrous and in the hair of horses [35,36]: this can explain the presence in donkey milk and consequently in cheese, where its concentration almost doubled. As regards cheese, 18 compounds were identified in the donkey product (versus 34 in the control one). The most abundant were acetoin, 2-methylbutanal and 2-ethylhexanol, followed by 2-ethylhexanal and hexanoic acid. Acetoin and methylbutanal are commonly found in any types of dairy products; they present butter and green odors and derive from lactic acid bacteria fermentation [37,38,39]. 2-Ethylhexanol has a fruity, green, cucumber odour [40] and likely derives from microbial metabolism, since it was not present in milk [41]. However, it has also been reported to be a pollutant deriving from plastic tools. In particular, it is a metabolic derivative of 2-ethylexylphtalate (DEHP), an additive commonly added to plastics to make them flexible [42]. Possible pollution from the cheesemaking environment (i.e., plastic electrode, tools for milking and plastic baskets used for cheese moulding) should be ascertained. All other newly formed compounds had been widely reported in the literature for cow’s cheese.

**Figure 3:**
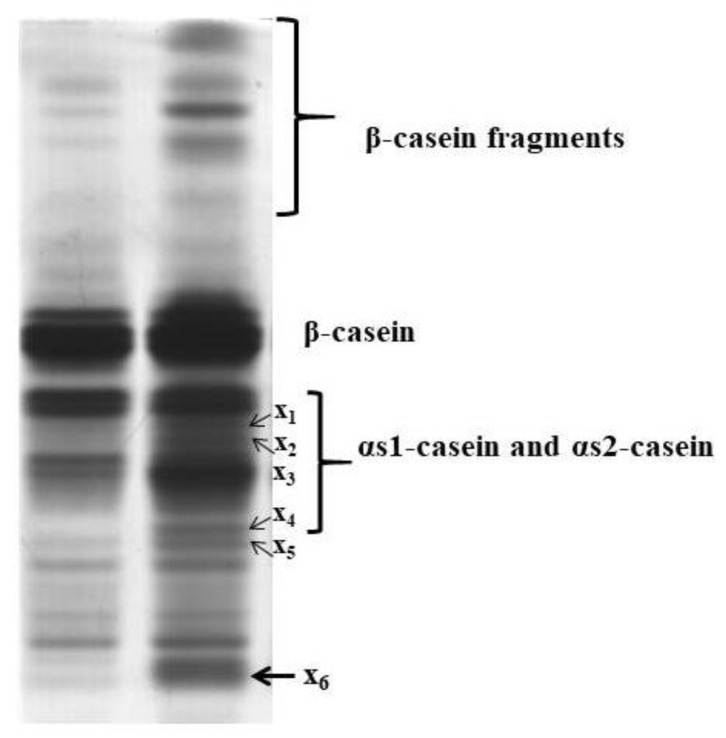
Urea polyacrylamide gel electrophoresis (urea-PAGE) patterns of donkey milk (left slot) and cheese after moulding (right slot).

**Figure 4:**
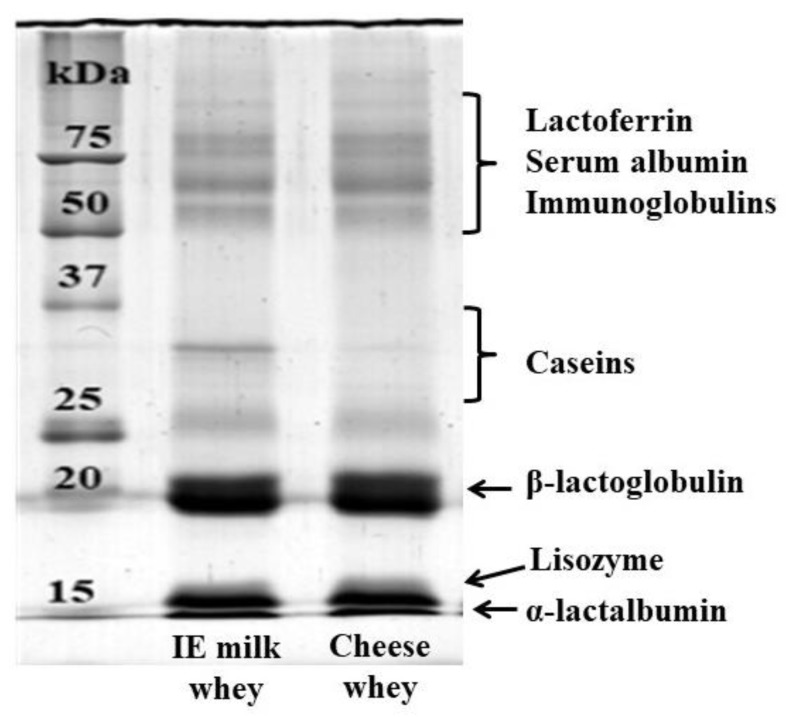
Sodium dodecyl sulphate (SDS)-PAGE patterns of isoelectric donkey milk whey (left slot) and donkey cheese whey after moulding (right slot).

Figure 3 and Figure 4 show the results of the electrophoretic study. For interpreting the gel, the works of Chianese et al. [12] on donkey milk, and of Egito et al. [19,20] on equine caseins in buffered solutions, were considered. By comparing the urea-PAGE patterns of milk and cheese (Figure 3), it was evident that some new bands appeared after 24 h from cheesemaking. In the upper zone of the gel, the intensity of several bands strongly increased in cheese: they should correspond to γ-casein-like fragments deriving from β-caseins. In the central area, five new bands appeared: for those coded x_1_ and x_2_, no possible correspondence was found in the literature, whereas those coded x_3_, x_4_ and x_5_ laid in the same zone of β-I- and β-II-caseins-like fragments. Finally, the diffuse band positioned in the lower zone (coded x_6_ and probably composed of two or three overlapping bands), could correspond to proteose-peptone-like fragments deriving from β-casein, or to fragments with high electrophoretic mobility deriving from αs1-casein. Considering that the cheese was analysed at 24 h after production, it is likely that these fragments derive from the activity of microbial rennet, which is able to hydrolyse all casein fractions in very short time [43].

Due to scarce resolution of whey proteins in the urea-PAGE system, SDS-PAGE was used for analysing the whey samples [44]. The patterns of isoelectric and cheese whey were very similar, except for the total absence of residual casein in the latter (Figure 4).

**Figure 5:**
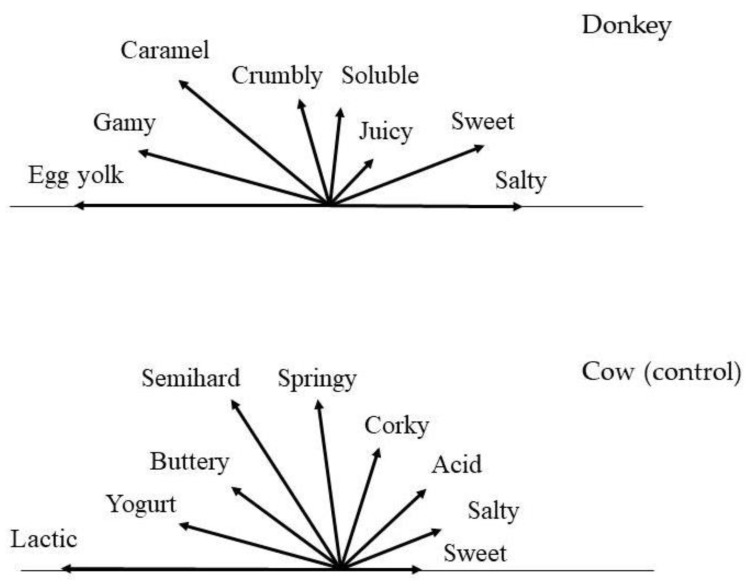
Flavour profile (aroma, texture and taste) of donkey and control cheese made with microbial rennet.

Table 4 and Figure 5 show the results of the sensory analyses. The restricted list of descriptors and the corresponding reference products that were developed for the experimental and control cheeses are shown in Table 4. For the former, 9 descriptors were selected from a pool of 13 initial attributes, 3 of which regarded texture, 3 aroma, 2 taste and 1 colour. The term “gamy” was adopted to indicate the typical animal odour of donkey milk. For the control, the descriptors were mostly different (10 developed). The colour was white in both cases, but with different gradations. The texture was very close to that of fresh Feta cheese for donkey, and to that of Edam cheese for cow. 

An extremely distinctive aroma was perceived in the former, for which a unique descriptor was developed, that is “egg yolk”. As to taste, the most intense descriptor selected was “sweet”, whereas the control also had a slight acid taste. This difference should be ascribed both to different pH values (it was slightly lower in the control cheese) and/or possible masking of the sour taste by the sweetness of donkey samples.

The differences in aroma, texture and taste can be clearly observed in Figure 5, where the flavour profiles are shown. The most intense odour in donkey cheese was “egg yolk”, followed by “gamy” and “caramel”. By comparing these results with those reported in the literature for egg yolk [45], a possible connection with the VOC profiles can be hypothesised. In fact, the two matrices present several volatile compounds in common, such as dimethyl sulphone, acetic, butanoic and hexanoic acids. As to texture, the cheese lacked elasticity, and became dry and chalky after a few days storage at 13 °C and 75% relative humidity, indicating that it was not suitable to ripening (results not shown). Overall, the sensory profile was rather similar to that previously reported for cheesemaking with calf rennet, and the flavour was judged as pleasant.

## 4. Conclusions

The present experimentation confirmed that donkey milk can be processed into fresh cheese by enzymatic coagulation under suitable technological conditions, by using microbial rennet. Such conditions result in being very severe when applied to cow milk, and lead to the formation of a semihard curd in a very short time. The chemical and sensory composition of fresh donkey cheese can be very interesting from a nutritional point view, due to very low fat levels and pleasant flavour. However, the scarce availability and high cost of the raw material currently limit practical application. Further studies are necessary by animal husbandry scientists to increase the milk yield of lactating donkeys, and by food scientists for improving the cheese texture and cheesemaking yield. Efforts for improving curd firmness should focus on increasing casein concentration: to this aim, protein enrichment could be pursued by water evaporation (at low temperature, in order to avoid deterioration of the already weak coagulation properties) or membrane filtration.

## Figures and Tables

**Table 1:** Chemical characteristics of donkey and cow milk and corresponding cheeses (mean values ± standard deviation (SD)).

Item	Donkey		Cow (Control)	
	Milk	Cheese	Milk	Cheese
pH	7.01 ± 0.03 ^a^	5.85 ± 0.10 ^c^	6.71 ± 0.02 ^b^	5.61 ± 0.06 ^d^
Total Solids%	n.a.	34.19 ± 2.64 ^b^	n.a.	49.88 ± 1.16 ^a^
Protein%	2.21 ± 0.11 ^d^	25.87 ± 3.37 ^a^	3.33 ± 0.07 ^c^	17.29 ± 2.96 ^b^
Fat%	0.33 ± 0.08 ^d^	2.90 ± 0.11 ^c^	3.62 ± 0.39 ^b^	23.76 ± 3.11 ^a^
Lactose%	6.76 ± 0.07 ^a^	n.a.	4.91 ± 0.02 ^b^	n.a.
Ash%	0.39 ± 0.06 ^d^	3.31 ± 0.30 ^b^	0.81 ± 0.05 ^c^	4.83 ± 0.44 ^a^
Total processing time *, min		42.10 ± 6.82 ^a^		13.20 ± 0.20 ^b^
Cheese yield%		7.21 ± 0.76 ^b^		23.52 ± 1.50 ^a^

Values in the same row bearing different superscripts (^a,b,c,d^) are significantly different, *p* < 0.05. n.a., not assessed; * from addition of rennet.

**Table 2:** Fat content (%) and fatty acid composition (% of total fatty acids) of donkey’s and cow’s milk, cheese and whey.

Item	Donkey			Cow (Control)		
	Milk	Cheese	Whey	Milk	Cheese	Whey
Total fat content	0.33 ± 0.08	2.90 ± 0.11	<0.1	3.62 ± 0.39	23.76 ± 3.11	0.28 ± 0.10
Butyric Acid	0.22 ± 0.11	0.19 ± 0.09	n.d.	4.99 ± 0.05	4.85 ± 0.11	5.19 ± 0.23
Caproic Acid	0.40 ± 0.21	0.72 ± 0.66	n.d.	2.73 ± 0.21	2.91 ± 0.19	2.03 ± 1.21
Caprylic Acid	3.69 ± 1.27 ^a^	1.50 ± 0.19 ^b^	2.71 ± 0.90 ^a^	1.29 ± 0.24	1.44 ± 0.34	1.51 ± 0.54
Capric Acid	7.85 ± 1.22 ^a^	4.08 ± 0.10 ^b^	6.00 ± 1.73 ^a^	2.41 ± 0.19	2.55 ± 0.49	2.92 ± 0.55
Undecanoic Acid	1.02 ± 0.22 ^a^	0.30 ± 0.12 ^b^	1.80 ± 0.64 ^a^	0.81 ± 0.11	0.73 ± 0.15	0.33 ± 0.39
Lauric Acid	7.56 ± 0.40 ^a^	4.07 ± 0.09 ^b^	6.74 ± 1.78 ^a^	4.21 ± 0.58	4.40 ± 0.27	4.33 ± 0.61
Tridecanoic Acid	0.03 ± 0.06	n.d.	n.d.	n.d.	n.d.	n.d.
Myristic Acid	6.07 ± 0.62 ^b^	12.16 ± 0.33 ^a^	7.77 ± 4.77 ^a,b^	11.94 ± 0.31	12.41 ± 0.77	13.07 ± 1.00
Myristoleic Acid	0.29 ± 0.07 ^b^	1.15 ± 0.46 ^a^	n.d.	0.49 ± 0.11	0.32 ± 0.21	0.44 ± 0.33
Pentadecanoic Acid	0.29 ± 0.37 ^b^	1.19 ± 0.46 ^a^	n.d.	0.90 ± 0.23	0.70 ± 0.31	0.61 ± 0.42
Palmitic Acid	19.08 ± 0.34 ^b^	33.08 ± 0.60 ^a^	21.37 ± 1.47 ^b^	28.45 ± 0.21	30.18 ± 0.37	29.41 ± 3.17
Palmitoleic Acid	2.27 ± 0.11 ^a^	1.97 ± 0.08 ^b^	2.48 ± 1.66 ^a,b^	1.37 ± 0.05	1.01 ± 0.49	1.78 ± 0.52
Margaric Acid	0.29 ± 0.20	0.71 ± 0.26	n.d.	0.45 ± 0.02	0.38 ± 0.12	n.d.
Eptadecenoic Acid	0.34 ± 0.46	n.d.	n.d.	n.d.	n.d.	n.d.
Stearic Acid	1.87 ± 0.47 ^c^	8.80 ± 0.15 ^a^	5.18 ± 3.14 ^b^	10.46 ± 0.17 ^b^	13.09 ± 1.22 ^a^	9.67 ± 0.94 ^b^
Elaidic Acid	n.d.	0.95 ± 0.16	n.d.	0.84 ± 0.30	0.54 ± 0.23	0.89 ± 0.21
Oleic Acid	24.46 ± 1.96	21.84 ± 2.01	24.34 ± 0.84	23.61 ± 0.91	20.08 ± 1.88	22.57 ± 1.90
Linoleic Acid	18.27 ± 0.25 ^a^	5.10 ± 0.13 ^b^	16.54 ± 3.46 ^a^	3.05 ± 0.56	2.80 ± 0.41	3.56 ± 0.68
Linolenic Acid	5.32 ± 0.16 ^a^	1.45 ± 1.66 ^b^	5.07 ± 2.56 ^a,b^	1.21 ± 0.11	0.97 ± 0.05	1.69 ± 0.97
Arachidic acid	0.09 ± 0.05 ^b^	0.21 ± 0.05 ^a^	n.d.	0.11 ± 0.04	0.23 ± 0.09	n.d.
Gadoleic Acid	0.24 ± 0.16	0.52 ± 0.46	n.d.	0.30 ± 0.11	0.41 ± 0.15	n.d.
Eicosadienoic Acid	0.29 ± 0.07	n.d.	n.d.	0.38 ± 0.01	n.d.	n.d.
Mean saturated	48.46	67.01	51.57	70.35	73.87	68.27
Mean monounsaturated	27.60	26.43	26.82	26.61	21.95	25.68
Mean polyunsaturated	23.92	6.55	21.61	4.64	4.18	5.25

Values in the same row bearing different superscripts (^a,b,c,d^) are significantly different, *p* < 0.05. n.d., not detected.

**Table 3:** Volatile organic compounds (VOCs) detected in milk and cheese (µg/kg ± SD).

Compoun.d.	Donkey		Cow (Control)	
	Milk	Cheese	Milk	Cheese
Acids				
acetic	5.65 ± 0.03 ^b^	0.19 ± 0.01 ^d^	2.69 ± 0.17 ^c^	66.03 ± 5.74 ^a^
butanoic	n.d.	0.36 ± 0.03 ^c^	5.09 ± 0.24 ^b^	8.77 ± 1.05 ^a^
hexanoic	n.d.	0.55 ± 0.10 ^c^	7.55 ± 0.87 ^b^	10.73 ± 0.82 ^a^
heptanoic	n.d.	n.d.	n.d.	0.68 ± 0.09
octanoic	n.d.	0.29 ± 0.07 ^c^	3.91 ± 0.33 ^b^	5.02 ± 0.22 ^a^
nonanoic	n.d.	n.d.	1..44 ± 0.03 ^b^	2.10 ± 0.07 ^a^
decanoic	n.d.	n.d.	3.22 ± 0.40	3.01 ± 0.44
Alcohols				
ethanol	n.d.	n.d.	n.d.	17.46 ± 2.07
2-ethylhexanol	n.d.	7.16 ± 0.12	n.d.	n.d.
isoamylalcohol	n.d.	0.42 ± 0.02	n.d.	n.d.
3-methyl,1-butanol	n.d.	n.d.	n.d.	71.84 ± 6.47
3-methyl-2-buten-1-ol	n.d.	n.d.	n.d.	1.19 ± 0.52
1-nonanol	n.d.	0.16 ± 0.01	n.d.	n.d.
2-octyloxy-ethanol	n.d.	n.d.	n.d.	16.26 ± 3.62
phenylethyl alcohol	n.d.	n.d.	n.d.	1.92 ± 0.09
2-propyl-1-pentanol	n.d.	n.d.	1.48 ± 0.39 ^a^	0.47 ± 0.16 ^b^
Aldheydes				
diacetyl (2,3-butanedione)	n.d.	n.d.	n.d.	27.35 ± 2.70
hexanal	n.d.	n.d.	1.04 ± 0.05 ^b^	5.33 ± 0.19 ^a^
heptanal	n.d.	n.d.	0.27 ± 0.07 ^b^	1.02 ± 0.10 ^a^
octanal	n.d.	n.d.	0.48 ± 0.13 ^b^	1.77 ± 0.51 ^a^
nonanal	0.02 ± 0.01 ^d^	0.39 ± 0.08 ^c^	3.22 ± 1.26 ^b^	17.97 ± 3.97 ^a^
decanal	n.d.	n.d.	2.05 ± 1.11	2.54 ± 0.33
2-methylbutanal	0.08 ± 0.01	8.79 ± 0.37	n.d.	n.d.
3-methylbutanal	n.d.	0.15 ± 0.01	n.d.	63.66 ± 0.29
2-ethylhexanal	0.09 ± 0.11	0.71 ± 0.02	n.d.	n.d.
*Esters*				
ethylacetate	0.09 ± 0.01 ^b^	0.08 ± 0.03 ^b^	n.d.	17.83 ± 5.72 ^a^
1-butanol-3-methyl-acetate	n.d.	n.d.	n.d.	1.03 ± 0.58
*Ketones*				
acetone	2.84 ± 0.03 ^c^	0.65 ± 0.01 ^d^	59.11 ± 9.11 ^a^	31.55 ± 0.29 ^b^
2-butanone	0.03 ± 0.01 ^d^	0.14 ± 0.02 ^c^	4.21 ± 1.21 ^b^	13.78 ± 2.85 ^a^
2-heptanone	0.09 ± 0.02 ^d^	0.37 ± 0.01 ^c^	4.81 ± 0.93 ^b^	16.09 ± 3.74 ^a^
3-heptanone	0.07 ± 0.01	n.d.	n.d.	n.d.
2-nonanone			n.d.	4.37 ± 0.47
2-un.d.ecanone			n.d.	0.85 ± 0.23
3-hydroxy 2-butanone (acetoin)	0.07 ± 0.02 ^c^	11.93 ± 0.04 ^b^	n.d.	91.71 ± 23.70 ^a^
6-methyl-5-hepten-2-one	0.15 ± 0.04 ^d^	0.38 ± 0.05 ^c^	0.88 ± 0.29 ^b^	1.83 ± 0.18 ^a^
2,3 pentanedione	n.d.	n.d.	0.60 ± 0.38 ^b^	23.56 ± 6.20 ^a^
*Others*				
dimethyl sulfide	n.d.	n.d.	0.12 ± 0.07 ^b^	1.33 ± 0.40 ^a^
dimethyl sulfone	n.d.	0.26 ± 0.01 ^b^	1.97 ± 0.28 ^a^	1.83 ± 0.19 ^a^
hexane	n.d.	n.d.	1.27 ± 0.53 ^b^	2.98 ± 0.81 ^a^
heptane	n.d.	n.d.	0.77 ± 0.12 ^b^	7.20 ± 1.00 ^a^

Values in the same row bearing different superscripts (^a,b,c,d^) are significantly different, *p* < 0.05. n.d., not detected.

**Table 4:** List of descriptors and reference product developed for donkey cheese.

Descriptor	Reference Product	Donkey	Cow (Control)
*Colour*			
White		*+*	
Ivory white			*+*
*Texture*			
Corky	Rubber stopper		+
Crumbly	Feta cheese	+	
Semi-hard	Carrot		+
Soluble	Meringue	+	
Springy	Edam cheese		+
Juicy	Water melon	+	
*Aroma*			
Buttery	Butter		+
Caramel	Cooked sugar	+	
Egg yolk	Raw egg yolk	+	
Gamy	Fresh donkey milk	+	
Lactic	Fresh uncultured milk		+
Yogurt	Plain yogurt		+
*Taste*			
Salty	0.5% NaCl in water	+	+
Sweet	0.5% sucrose in water	+	+
Acid	0.5% citric acid		+
